# *In vitro* antibacterial activity of gepotidacin in combination with other antimicrobial agents against *Neisseria gonorrhoeae* isolates

**DOI:** 10.1128/aac.00059-26

**Published:** 2026-07-02

**Authors:** Laura M. Koeth, Jeanna DiFranco-Franco, Josh West, Nicole E. Scangarella-Oman

**Affiliations:** 1 Laboratory Specialists, Inc., Westlake, Ohio, USA; 2 Infectious Diseases Research Unit, GSK, Collegeville, Pennsylvania, USA; Shionogi Inc., Florham Park, New Jersey, USA

**Keywords:** antibacterial agent, antimicrobial susceptibility testing, gepotidacin, checkerboard assay, *N. gonorrhoeae*

## Abstract

This study was conducted to determine the potential effect of other antimicrobial agents on the in vitro activity of gepotidacin against six WHO *N. gonorrhoeae and* QC strain ATCC 49226 by checkerboard assays using Graver-Wade broth. Synergy and antagonism were evaluated using fractional inhibitory concentration (FIC) and FIC index (FICI) results for gepotidacin and comparator agents (azithromycin, cefixime, ceftriaxone, gemifloxacin, sitafloxacin, gentamicin, spectinomycin, doxycycline, and lefamulin). The evaluation of FICI showed no antagonistic or synergistic interactions, while the evaluation of individual FIC values revealed strain- and agent-specific synergistic or additive effects.

## INTRODUCTION

Gepotidacin is a novel triazaacenaphthylene antibiotic that selectively inhibits type IIA topoisomerases through a distinct binding site and novel mechanism of action, resulting in activity against drug-susceptible and -resistant/nonsusceptible *N. gonorrhoeae*, as well as other important pathogens (1–5). Gepotidacin received approval for the treatment of uncomplicated urinary tract infections in the United States and Europe, and for uncomplicated urogenital gonorrhea in the United States (6–9).

Antimicrobial resistance in *N. gonorrhoeae* has been well documented globally through surveillance, with rising azithromycin resistance, persistent fluoroquinolone resistance, and sporadic ceftriaxone-resistant strains (10–12). Antimicrobial combination testing is performed during drug development to identify potential antagonistic or synergistic interactions. The checkerboard broth microdilution method can serve as an initial screen, with time-kill assays used for more definitive assessment, followed by confirming synergy or antagonism *in vivo*.

We conducted checkerboard assays for initial screening of gepotidacin and nine antimicrobial agents using seven *N. gonorrhoeae*. The minimal inhibitory concentration (MIC) was determined by CLSI agar dilution (AD) since there is no broth microdilution (BMD) method for *N. gonorrhoeae* (13, 14). Checkerboard assays were based on CLSI BMD, except those using Graver-Wade (GW) medium, a broth developed for BMD testing of *N. gonorrhoeae* and also utilized for time-kill assays (15–17). Triplicate MICs of each antibiotic alone and in combination were used to calculate fractional inhibitory concentration (FIC) and FIC index (FICI) (FICI = FICI A + FICI B, where *FICI A* represents the MIC of antibiotic A in combination divided by the MIC of antibiotic A alone and *FICI B* represents the MIC of antibiotic B in combination divided by the MIC of antibiotic B alone). Modal FICI values were interpreted as synergistic when values were ≤0.5, additive/indifferent at >0.5–4.0, and antagonistic at >4 (15, 18). Additive effects show slight enhancement of activity (FICI >0.5–1), whereas indifferent effects show no change with combination (FICI >1–≤4). In addition, modal FIC values for both agents A and B were used as separate metrics and interpreted as synergistic at ≤0.25, additive/indifferent at >0.25–2.0, and antagonistic at >2 (19). When the MIC was greater than the highest concentration tested, the highest concentration plus one dilution was used, and when the combination MIC was less than the lowest concentration tested, the lowest concentration was used. The isolates used in this study were WHO strains (F, L, M, O, V, and X) as described by Unemo et al. (20) and quality control (QC) strain *N. gonorrhoeae* ATCC 49226 (14). These strains were chosen to encompass a range of gepotidacin MIC results (0.06–2 μg/mL) with specific drug resistance profiles. Gepotidacin was sourced from GSK, and comparator agents were sourced from TCI America (azithromycin), Sigma (cefixime, ceftriaxone, doxycycline, gemifloxacin, and gentamicin), and MedChem Express (lefamulin, sitafloxacin, and spectinomycin). Inoculum concentration was verified by colony counts sampled from the positive control well for one replicate of each isolate tested (recommended concentration/range of 5 × 10^5^/2–8 × 10^5^ CFU/mL) (13).

MIC and checkerboard testing yielded several notable findings. GW broth supported the growth of study isolates and provided adequate inoculum concentration (average 5.1 × 10^5^ and range 4.4–5.9 × 10^5^ CFU/mL) and clear determination of MICs. BMD MICs were within the concentrations tested (on scale) for 55/70 results; compared to AD results, 83.6% were within essential agreement (±1 dilution), and the majority of these (76.1%) were one dilution lower (Table 1). Similarly, for non-fastidious bacteria, BMD MICs are typically one dilution lower than AD MICs, possibly due to inherent methodological differences in growth kinetics and drug availability between liquid and solid media (13). CLSI AD QC ranges were available for five of the agents tested (gepotidacin, azithromycin, ceftriaxone, cefixime, and spectinomycin). All AD results were within established ranges. Subsequent to testing performed in this study, the gentamicin AD MIC range of 4–16 μg/mL was established by CLSI (21). Gentamicin AD results from this study were outside of this range (2 μg/mL). BMD QC results were equal to or one to two dilutions lower than AD QC results (where endpoints were achieved).

**TABLE 1:** Modal agar dilution and broth microdilution MIC results and dilution difference for gepotidacin and comparators against seven *N. gonorrhoeae* isolates*^a^*^,^*^b^*^,^^*c*^

Agent	Range of modal AD MIC (µg/mL)	Range of modal GW BMD MIC (µg/mL)	Dilution difference (modal BMD MIC – modal AD MIC)							WHO strain with BMD MIC >1 dilution from AD MIC
			OS <	−3	−2	−1	0	1	OS >	
Gepotidacin	0.25–4	0.06–2			2	5				F and X = −2
Azithromycin	0.06–>1024	0.03–>512			2	3		1	1	O and X = −2
Cefixime	0.004–8	≤0.008–4	3			3	1			
Ceftriaxone	≤0.002–2	≤0.008–1	4			2	1			
Gemifloxacin	0.004–4	≤0.002–8	2		1	1	2	1		O = −2
Sitafloxacin	≤0.016–0.5	≤0.002–0.25	3			4				
Gentamicin	2–8	1		2		5				V and X = −3
Spectinomycin	32–>1024	16–>512			1	5			1	
Doxycycline	1–4	0.5–2			1	2	4			F = −2
Lefamulin	0.12–2	≤0.06–1	1			5	1			

^*a*^ OS, off scale (lower than the lowest concentration or higher than the highest concentration tested).

^*b*^ AD, agar dilution; BMD, broth microdilution. Modal AD and BMD MIC results based on three replicates. Gepotidacin BMD modal MIC based on 27 replicates.

^*c*^ Gray shading indicates expected MIC variation of ±1 dilution.

FICI values determined by checkerboard BMD testing showed no antagonism or synergy, and all results were considered additive/indifferent or not evaluable, except for one replicate of WHO M tested against gepotidacin in combination with azithromycin, for which synergy was observed (Table 2). Modal gepotidacin FICs were interpreted as synergistic when tested with cefixime for five isolates, ceftriaxone for four isolates, gemifloxacin for three isolates, and sitafloxacin and spectinomycin for two isolates (Table 3). Modal FICs for the comparator agents tested with gepotidacin were interpreted as synergistic for azithromycin and doxycycline for four isolates, lefamulin for three isolates, gemifloxacin and sitafloxacin for two isolates, and cefixime, ceftriaxone, and spectinomycin for one isolate(s) (Table 3). All other FIC results were interpreted as additive/indifferent or not evaluable. The FICI and FIC calculations for determination of synergy require that the MIC is at least two dilutions lower for both agents, respectively. Since some results for specific agents and isolates were off scale at less than the lowest concentration tested, they were not evaluable (Tables 2 and 3).

**TABLE 2:** Evaluation of Antimicrobial activity based on FICI

Modal FICi based on three replicate results for each agent when tested with gepotidacin							
Agent	Isolate designation						
	F	L	M	O	V	X	QC
Azithromycin	1.03^*a*^	1.13	0.74^*b*^	0.98	1.01	1.27	1.06
Cefixime	1.5^*a*^	0.63	1.03^*a*^	1.13^*a*^	0.75^*a*^	1.25	0.74^*a*^
Ceftriaxone	1.5^*a*^	1.0	1.03^*a*^	1.13^*a*^	0.75^*a*^	1.25	1.13^*a*^
Doxycycline	1.5^*a*^	1.0	0.63	0.73	0.75	1.0	0.98
Gemifloxacin	1.5^*a*^	0.75	0.98	0.74^*a*^	0.75	0.98	1.13
Lefamulin	1.5^*a*^	0.98	0.74	0.98	1.0	0.98	1.24
Sitafloxacin	1.5^*a*^	0.98	0.75	1.13^*a*^	1.0	0.98	1.13^*a*^
Spectinomycin	1.0^*a*^	1.5^*a*^	1.0^*a*^	1.01	1.25^*a*^	0.75^*a*^	0.98^*a*^

^*a*^ Synergy based on the FICI is not evaluable since MIC results for the agent tested alone and/or in combination is ≤ the lowest concentration tested and both MIC results are within one dilution of each other.

^*b*^ FICI for one replicate was 0.3 (synergistic).

**TABLE 3:** Evaluation of antimicrobial activity based on individual agent FICs^*b*^

	Agent	Isolate designation						
		F	L	M	O	V	X	QC
Gepotidacin modal FIC results when tested with each agent	Azithromycin	0.5^*a*^	1	0.5	0.5	1	0.5	0.5
	Cefixime	0.5^*a*^	**0.125** (2, 0.25)	**0.03** (1, ≤ 0.03)	**0.125** (0.25, ≤ 0.03)	**0.25** (0.12, ≤ 0.03)	1	**0.25** (0.25, 0.06)
	Ceftriaxone	0.5^*a*^	0.5	**0.03** (1, ≤ 0.03)	**0.125** (0.25, ≤ 0.03)	**0.25** (0.12, ≤ 0.03)	1	**0.125** (0.25, 0.03)
	Doxycycline	1	0.5	0.5	0.5	0.5	0.5	0.5
	Gemifloxacin	0.5^*a*^	**0.25** (2, 0.5)	0.5	**0.25** (0.25, 0.06)	0.5	0.5	**0.125** (0.25, ≤ 0.03)
	Lefamulin	0.5^*a*^	0.5	0.5	0.5	0.5	0.5	1
	Sitafloxacin	0.5^*a*^	0.5	0.5	**0.125** (0.25, ≤ 0.03)	0.5	0.5	**0.125** (0.25, ≤ 0.03)
	Spectinomycin	0.5^*a*^	1	0.5	0.5	**0.25** (0.12, ≤ 0.03)	**0.25** (0.12, ≤ 0.03)	0.5
Modal FIC results for each agent when tested with gepotidacin	Azithromycin	0.5^*a*^	**0.25** (0.06, ≤ 0.016)	**0.25** (0.25, 0.06)	**0.25** (0.06, ≤ 0.016)	**0.008** (>512, 8)	0.5	0.5
	Cefixime	1^*a*^	0.5	1^*a*^	1^*a*^	0.5^*a*^	**0.25** (1, 4)	0.5^*a*^
	Ceftriaxone	1^*a*^	0.5	1^*a*^	1^*a*^	0.5^*a*^	**0.25** (1, 0.25)	1^*a*^
	Doxycycline	0.5^*a*^	**0.125** (2, ≤ 0.25)	**0.125** (2, ≤ 0.25)	**0.25** (2, 0.5)	**0.25** (2, 0.5)	0.5	0.5
	Gemifloxacin	1^*a*^	0.5	0.5	0.5^*a*^	**0.25** (2, 0.5)	**0.25** (2, 8)	1^*a*^
	Lefamulin	1^*a*^	0.5	**0.25** (0.25, ≤ 0.06)	0.5	**0.25** (1, 0.25)	0.5	**0.25** (0.25, ≤ 0.06)
	Sitafloxacin	1^*a*^	0.5	**0.25** (0.12, 0.03)	1^*a*^	**0.25** (0.12, 0.03)	0.5	1^*a*^
	Spectinomycin	0.5^*a*^	0.5^*a*^	0.5^*a*^	**0.25** (>512, 256)	0.5^*a*^	0.5^*a*^	0.5^*a*^

^*a*^ Synergy based on the FIC is not evaluable since MIC results for the agent tested alone and/or in combination are ≤ the lowest concentration tested, and both MIC results are within one dilution of each other.

^*b*^ If FICs are ≤0.25, they are shown in bold print followed by *(*modal MIC tested alone, modal MIC tested with other agent*)***.**

Interpretation of combination testing data requires consideration of several methodological constraints and limitations observed in this study. Since the strains and the concentration of agents tested in some cases did not provide for on-scale MIC results (especially at low concentrations), FIC and FICI results were not evaluable. In these cases, since the MICs were very low, detection of synergy was not necessarily relevant, and in all cases, no antagonism was observed. For further testing, however, plate concentrations specific to each strain/agent should be used, provided they are within accurate/precise levels for the test agent(s). In order to better evaluate synergy, since the majority of strains tested were susceptible to gepotidacin and the comparator agents, any further testing should include more isolates with elevated MICs (if available). There have been relatively few published studies recently of synergy studies for *N. gonorrhoeae*, and standardization of synergy methods is needed. Variability in the media used for one other checkerboard study and the use of gradient diffusion methods in two other studies limit the interpretability of FIC and FICI values and constrain how confidently our findings can be compared (22–24). Since GW broth supported the growth and endpoints were easy to read in this study, additional studies comparing GW broth checkerboard to GW broth time-kill assays are warranted. In order to address any impact on the bias of slightly lower BMD results in comparison to AD results, further evaluation of broth checkerboard results based on the correlation to *in vivo* efficacy and PK/PD factors should be considered.

Taken together, the FICI results indicate that gepotidacin shows no antagonistic or synergistic interactions with the antimicrobials tested. However, when gepotidacin and the other drugs are evaluated separately by FIC results, synergistic and additive effects were observed.
